# Deciphering the link between healthcare expenditure, corruption, and COVID-19 mortality

**DOI:** 10.1038/s41598-024-63766-6

**Published:** 2024-06-03

**Authors:** Jay Squalli

**Affiliations:** 1 Department of Economics, American University of Sharjah, Sharjah, United Arab Emirates; 2 Center for Entrepreneurship, Innovation and Sustainable Development, American University of Sharjah, Sharjah, United Arab Emirates

**Keywords:** Environmental economics, Socioeconomic scenarios, Viral infection

## Abstract

This paper analyzes the determinants of COVID-19 mortality across over 140 countries in 2020, with a focus on healthcare expenditure and corruption. It finds a positive association between COVID-19 deaths and aging populations, obesity rates, and healthcare expenditure while noting a negative association with rural residency and corruption perception. The study further reveals that mortality is positively associated with aging populations in high-income countries and positively associated with obesity in upper-middle to high-income countries. Mortality is positively associated with healthcare expenditure, which likely reflects a country’s preparedness and ability to better track, document, and report COVID-19 deaths. On the other hand, mortality is negatively associated with corruption perception in upper-middle-income countries. Further analyses based on 2021 data reveal COVID-19 deaths are positively associated with the proportion of the population aged 65 and older in low to lower-middle-income countries, with obesity in high-income countries, and with tobacco use across most countries. Interestingly, there is no evidence linking COVID-19 deaths to healthcare expenditure and corruption perception, suggesting a post-2020 convergence in preparedness likely due to proactive pandemic responses, which might have also mitigated corruption’s impact. Policy recommendations are proposed to aid the elderly, address obesity, and combat tobacco use.

## Introduction

The COVID-19 pandemic has had a devastating and far-reaching impact on communities around the world, resulting in millions of deaths, disrupting daily life, and sending shockwaves through global economies. While the effect of the pandemic may have dissipated to a great extent, COVID-19 continues to affect people worldwide. There are still cases, hospitalizations, and deaths occurring, particularly in areas with low vaccination rates or because of new variants of the virus.

Lessons learned from the COVID-19 pandemic and especially from the year 2020 hold particular significance. This period witnessed the peak of COVID-19 mortality worldwide, laying bare the unpreparedness of many countries to face such a public health crisis. Healthcare systems were overwhelmed and societies grappled with unprecedented challenges as they struggled to contain the spread of the virus. Various countries responded differently to the crisis, with notable examples such as New Zealand demonstrating effective strategies in managing the pandemic^1^, and the United States facing significant challenge^2^.

The effectiveness of the measures adopted by various nations to combat the pandemic is contingent upon factors that can only be identified and assessed during the nascent stages. For instance, the readiness and resilience of healthcare systems vary widely across countries. Factors such as healthcare expenditure, availability of medical supplies, hospital capacity, and healthcare workforce can significantly impact the ability to detect, diagnose, treat, and contain the virus. Thus, analyzing data beyond 2020 may not fully capture important characteristics about countries that could be obscured by the measures adopted to deal with the pandemic. For example, countries may have implemented emergency healthcare policies, redirected resources, or adopted temporary measures that may not be reflective of their healthcare infrastructure or preparedness.

Understanding the factors contributing to COVID-19 outcomes during the year 2020 can help shed light on how to develop better strategies for managing and mitigating the impact of new infectious diseases that may arise in the future. Central to this goal is understanding the determinants of mortality, which could equip healthcare systems to respond to ongoing challenges and prepare more effectively for future pandemics. For instance, one of the most important lessons is the fact that the pandemic has highlighted health disparities and the disproportionate impact of the virus on vulnerable populations, such as the elderly and those with underlying health conditions. By gaining further insights into the determinants of mortality, we can drive efforts to address these disparities and enhance healthcare access and outcomes for all. This would also play a crucial role in helping policymakers make better-informed decisions regarding public health measures, vaccination campaigns, and the allocation of healthcare resources.

To date, our understanding of the determinants of COVID-19 deaths has been enriched by a substantial body of research. Various factors potentially contributing to COVID-19-related deaths have been identified, including being aged 65 and older^3–5^, comorbidities^4–6^, smoking status,^5,7^ lower education attainment^7^, air pollution^7–14^, and living in rural areas^15,16^, among other contextual factors. This study builds upon existing research by exploring additional dimensions that may have substantial implications for understanding the determinants of COVID-19 mortality, namely healthcare expenditure and corruption.

Two hypotheses are proposed for the link between healthcare expenditure and COVID-19 deaths. First, increased healthcare expenditure, when efficiently directed toward enhancing healthcare infrastructure, workforce, prevention, testing, and treatments, is hypothesized to result in lower COVID-19 deaths. This suggests that well-funded and efficient healthcare systems with ample resources can deliver timely and effective care, curb virus transmission, and mitigate the severity of COVID-19 cases, ultimately reducing mortality rates. However, this also suggests that even with substantial expenditure, an inefficient and poorly organized healthcare system may struggle with curbing virus transmission and severity, thereby resulting in higher mortality. Second, a country’s healthcare expenditure may measure a country’s effectiveness in tracking and accurately reporting COVID-19 deaths. This hypothesis assumes that nations with higher healthcare expenditure relative to their GDP may possess superior healthcare infrastructure and surveillance systems, potentially leading to more precise diagnosis, tracking, and reporting of COVID-19 fatalities. As a result, this hypothesis implies a positive relationship between healthcare expenditure and reported COVID-19 deaths.

In terms of the link between corruption and COVID-19 deaths, two hypotheses are put forth. First, a country’s corruption rate may indicate a nation’s potential to misreport COVID-19 deaths. This hypothesis is grounded in the idea that countries with higher corruption rates may be more susceptible to data manipulation or underreporting for various motives, including reputational, political, or economic interests. This suggests a negative association between corruption and COVID-19 mortality. Second, countries with heightened levels of corruption may divert healthcare resources away from pandemic response efforts, resulting in inadequate diagnosis, testing, treatment, and curbing virus transmission, thereby resulting in increased COVID-19 mortality. This would suggest a positive association between corruption and COVID-19 mortality.

The link between healthcare expenditure and COVID-19 mortality has been the subject of a small number of studies, conducted in various contexts and yielding mixed results. The first study on this link made use of COVID-19 mortality data up to May 4, 2020 for more than 140 countries and provided evidence of a positive relationship^17^. Another study focusing on the European Union used cumulative data up to the second week of January 2021 confirmed these findings^18^. However, a cross-country analysis for 161 countries presumably using data up to December 14, 2020 provided evidence of a negative relationship^13^. Furthermore, there is just one study that analyzed the link between corruption and COVID-19 mortality^19^. This study used data for 91 countries for the year 2020 and provided evidence of a positive relationship.

The present study seeks to contribute to the existing literature by utilizing data spanning the entirety of 2020 for COVID-19 deaths and a sample of up to 159 countries. In addition, it integrates more recent data from 2019 for other pertinent variables, such as healthcare spending and corruption, providing a more precise portrayal of the conditions of these factors in various countries during the early stages of the pandemic. This approach stands out as an improvement over prior studies, which utilized data for earlier periods and partial data on COVID-19 deaths in 2020. In particular, this research aims to fill research gaps by focusing on immediate pandemic outcomes, which could overshadow pre-existing disparities or weaknesses in healthcare systems that may have contributed to varying mortality. The findings of this study can guide policymakers, healthcare professionals, and global health organizations in devising effective strategies designed to mitigate the impact of a similar pandemic and to enhance transparency in reporting pandemic-related data. To this end, this paper is organized as follows: "Methods" section describes the data and methodology, "Results" section summarizes the results, and "Discussion" section discusses the results and concludes.

## Methods

### Data

The data used in this study are comprised of total population, the proportion of individuals aged 65 and above, the percentage of rural population relative to the total population, prevalence of tobacco use among adults, mean annual exposure to particulate matter 2.5 (measured in micrograms per cubic meter, $\upmu g$/${\text m}^3$), government expenditure on education as a percentage of GDP, and current healthcare expenditure as a percentage of GDP, which were obtained from the World Bank’s World Development Indicators. For data related to COVID-19 deaths, representing the cumulative number of fatalities up to December 31, 2020, and data regarding the prevalence of obesity among adults (defined as having a Body Mass Index (BMI) greater than or equal to 30), we relied on data provided by the World Health Organization (WHO). Lastly, data on corruption were sourced from Transparency International. All data are from 2019 and the variables and their relevant details are described in Table 1. Corresponding summary statistics are presented in Table 2.

**Table 1:** Description of the variables.

Variable	Description	Source	Year
logdeaths	ln of number of deaths	World Health Organization	2020
logpop	ln of population	World Bank WDI	2019
pop65plus	% of population aged 65+	World Bank WDI	2019
obesity	Prevalence of obesity among adults (%)	World Health Organization	2019
ruralpop	Rural population (% of total population)	World Bank WDI	2019
tobacco	Prevalence of current tobacco use (% of adults)	World Bank WDI	2019
logpm25	ln of PM2.5 air pollution,	World Bank WDI	2019
	mean annual exposure ($\upmu g$/${\text m}^3$)		
education	Government expenditure on education (% of GDP)	World Bank WDI	2019
healthcare	Current health expenditure (% of GDP)	World Bank WDI	2019
corruption	Corruption perceptions Index	Transparency International	2019

**Table 2:** Summary statistics.

Variable	Obs	Mean	Std. Dev.	Min	Max
logdeaths (2020)	174	6.403	2.752	0.693	12.771
logpop (2019)	200	15.326	2.449	9.302	21.065
pop65plus (2019)	200	9.196	6.606	1.172	35.621
obesity (2019)	186	22.224	12.941	1.57	74.37
ruralpop (2019)	200	38.831	23.668	0	86.75
tobacco (2019)	157	20.461	9.7	3.6	49.2
logpm25 (2019)	191	3.048	0.685	1.717	4.421
education (2019)	168	4.49	2.161	9.05e-06	15.75
healthcare (2019)	177	6.512	3.06	1.532	20.89
corruption (2019)	169	43.361	19.045	9	87

It is worth noting that endogeneity is not a significant concern in this paper. The temporal distinction in the data, with the explanatory variables captured during years preceding COVID-19 deaths, significantly reduces the risk of endogeneity. In particular, the exclusion of response variables such as vaccination and lockdown, which are prone to high endogeneity, further strengthens this argument^19^. The data used in this study essentially capture predetermined characteristics that delineate the state of countries prior to the onset of the pandemic. This temporal distinction ensures that the identified associations between explanatory variables and COVID-19 mortality remain robust and are less susceptible to biases arising from endogeneity.

### Methodology

To capture the numerous factors that can contribute to COVID-19 mortality, it is important to also control for factors contributing to mortality in the absence of COVID-19. Not accounting for such factors could result in erroneously attributing COVID-19 deaths to some hypothesized factors.

The following represents a specification based on our current understanding of the COVID-19 virus and related hypotheses:

1 $$\begin{aligned} \text{ logdeaths}_i= & {} \alpha _{0}+\alpha _{1} \text{ logpop}_i+\alpha _{2} \text{ pop65plus}_{i}+ \alpha _{3} \text{ obesity}_{i}+\alpha _{4} \text{ ruralpop}_{i} \nonumber \\{} & {} +\alpha _{5} \text{ tobacco}_{i}+\alpha _{6} \text{ logpm25}_{i}+\alpha _{7}\text{ education}_{i}+\alpha _{8} \text{ healthcare}_{i} \nonumber \\{} & {} + \alpha _{9} \text{ corruption}_{i}+ \epsilon _{i} \end{aligned}$$

where the dependent variable represents the natural logarithm of the number of reported COVID-19 deaths (logdeaths) for country *i*. This is estimated as a function of the natural logarithm of total population (logpop), the share of the population above 65 years of age (pop65plus), the prevalence of obesity (obesity), the proportion of the total population living in rural areas (ruralpop), the prevalence of tobacco use (tobacco), the natural log of particulate matter 2.5 mean annual exposure, percent government expenditure on education (education), percent current healthcare expenditure (healthcare), and the corruption perception index (corruption). Table 3 provides a correlation matrix of the independent variables. This table shows correlations that are low enough to allay concerns about multicollinearity,instability of estimates, and overfitting.

**Table 3:** Correlation Matrix of Independent Variables.

Variables	logpop	pop65plus	obesity	ruralpop	tobacco	logpm25	education	healthcare	corruption
logpop	1.000								
pop65plus	− 0.087	1.000							
obesity	− 0.2854	0.2125	1.000						
ruralpop	0.117	− 0.447	− 0.5458	1.000					
tobacco	− 0.128	0.376	0.0775	− 0.088	1.000				
logpm25	0.431	− 0.585	− 0.2519	0.381	− 0.352	1.000			
education	− 0.206	0.007	0.1048	− 0.122	0.127	− 0.279	1.000		
healthcare	− 0.158	0.383	0.2460	− 0.306	0.235	− 0.518	0.408	1.000	
corruption	− 0.225	0.629	0.2526	− 0.465	0.189	− 0.546	0.359	0.418	1.000

Since the dependent variable is measured in levels, it is crucial to introduce the population as a scale variable. In addition, all variables not expressed in percentages are log-transformed to facilitate the interpretation of parameter estimates as elasticities. The *pop65plus* variable is introduced as an explanatory variable, in line with previous research suggesting a significant proportion of COVID-19 fatalities are individuals aged 65 and above^3–5^. The *obesity* variable is included to account for the established contribution of obesity to mortality^5^. The *ruralpop* variable addresses the disparities in mortality between urban and rural areas^15,16^. The *tobacco* variable factors in the relationship between smoking prevalence and COVID-19 mortality^5,7^. The *education* variable controls for the effect of lower education on mortality^7^. Lastly, the *logpm25* variable controls for the impact of air pollution on mortality.Previous research has consistently presented evidence supporting a positive relationship between air pollution and COVID-19 mortality^7–14^.

The model also incorporates the two variables of interest: *healthcare* and *corruption*. The *healthcare* variable is used to test hypotheses regarding the association between healthcare expenditure and COVID-19 mortality, as suggested by previous research^13,17,18^. On the other hand, the *corruption* variable is included to assess the association between corruption and mortality, a variable that has received little attention^19^. A higher corruption perceptions index implies lower corruption level.

Stepwise regression is used in this study for selecting a parsimonious specification with fewer variables for interpretation. This iterative process of model refinement begins with a stepwise forward selection approach, which involves sequentially adding predictors based on their potential to contribute significantly to explaining the variation in COVID-19 deaths. Following the forward selection step, a backward elimination approach is employed by systematically removing the least statistically significant predictors, ensuring that only the most robust variables remain. These steps allow the model to achieve a balance between complexity and explanatory power. The resulting specification with fewer variables not only yields a more interpretable model but also mitigates the risk of overfitting.

All estimations are completed using a Least Squares estimator with bootstrapped standard errors. This estimator allays concerns about within-sample distortions and derives estimates of standard errors and confidence intervals based on the underlying distribution of the sample rather than based on some a priori distributional assumptions. All estimations are completed with 500 bootstrap replications.

**Figure 1:**
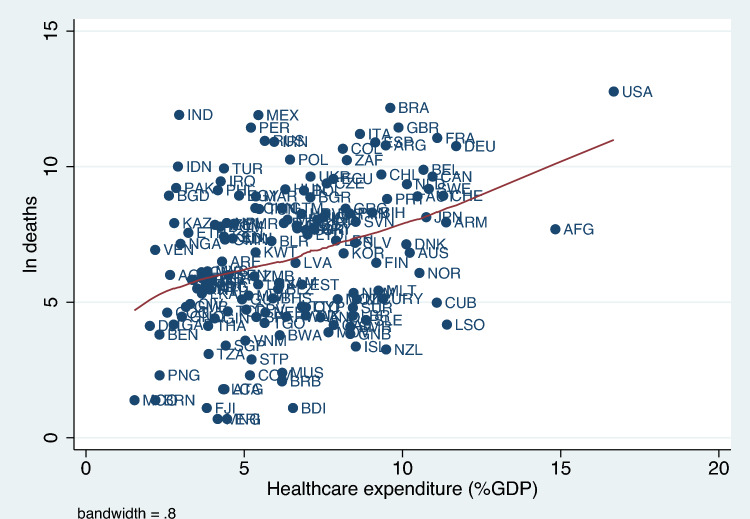
Plot of ln deaths and healthcare expenditure.

**Figure 2:**
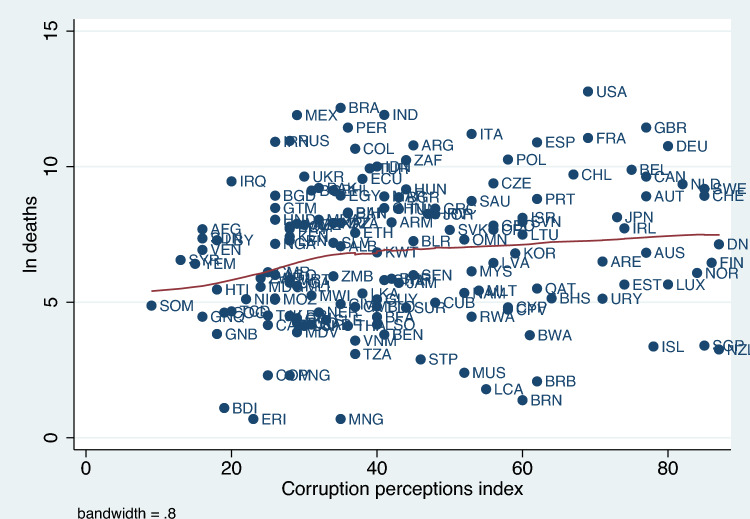
Plot of ln deaths and corruption perceptions index.

In a preliminary analysis of the hypothesized relationships, scatter plots are derived using the locally weighted scatterplot smoother (lowess). This approach is preferable over a simple linear regression line because it offers more flexibility by not assuming a specific functional form for the relationship between variables^20^. As a result, it can capture complex patterns in the data without imposing rigid assumptions. Figures 1 and 2 illustrate the associations between healthcare spending and COVID-19 mortality and between corruption and mortality, respectively. At first glance, these figures appear to reveal a positive relationship. However, the following section will further investigate these relationships by accounting for other potential confounding factors, aiming to validate the patterns observed in the figures.

## Results

The estimation results presented in Table 4 are derived from a stepwise regression, a method that incrementally introduces variables to identify the model with the greatest explanatory power. The initial model in column (1) indicates that the coefficients for *logpop* and *pop65plus* are both positive and statistically significant ($p<0.001$). After introducing *obesity* in column (2), the coefficients for *logpop* and *pop65plus* are unaffected while the coefficient for *obesity* is also positive and statistically significant ($p<0.001$).

**Table 4:** Bootstrap estimation results (2020).

	(1)	(2)	(3)	(4)	(5)	(6)	(7)	(8)	(9)
logpop	0.950***	1.091***	1.082***	1.065***	1.039***	1.025***	1.014***	1.012***	1.045***
	(0.0608)	(0.0712)	(0.0692)	(0.0854)	(0.0860)	(0.0881)	(0.0904)	(0.0887)	(0.0737)
pop65plus	0.149***	0.137***	0.118***	0.107***	0.131***	0.121**	0.0946**	0.123**	0.126***
	(0.0213)	(0.0172)	(0.0176)	(0.0275)	(0.0367)	(0.0395)	(0.0349)	(0.0402)	(0.0245)
obesity		0.0939***	0.0806***	0.0864***	0.0878***	0.0788***	0.0756***	0.0750***	0.0809***
		(0.0114)	(0.0130)	(0.0167)	(0.0185)	(0.0166)	(0.0179)	(0.0185)	(0.0136)
ruralpop			− 0.0143*	− 0.0141	− 0.0145	− 0.0179	− 0.0184*	− 0.0227*	− 0.0190**
			(0.00680)	(0.00831)	(0.00957)	(0.00953)	(0.00931)	(0.00954)	(0.00718)
tobacco				0.0158	0.0148	0.0109	0.0167	0.0109	
				(0.0189)	(0.0182)	(0.0204)	(0.0182)	(0.0188)	
logpm25					0.353	0.314	0.417	0.240	
					(0.305)	(0.370)	(0.361)	(0.326)	
education						0.00821	− 0.0305	0.0136	
						(0.130)	(0.142)	(0.160)	
healthcare							0.133	0.123	0.119*
							(0.0704)	(0.0672)	(0.0529)
corruption								− 0.0241	− 0.0232*
								(0.0124)	(0.0100)
Intercept	− 9.976***	− 14.05***	− 12.91***	− 12.91***	− 13.81***	− 13.01***	− 13.64***	− 12.04***	− 11.91***
	(0.992)	(1.214)	(1.302)	(1.554)	(1.812)	(2.223)	(2.118)	(2.148)	(1.435)
Adj. $R^{2}$	0.601	0.672	0.680	0.656	0.658	0.627	0.636	0.645	0.691
$N$	174	163	163	141	141	129	127	124	150

Standard errors in parentheses

*$$p<0.05$$, **$$p<0.01$$, ***$$p<0.001$$.

Column (3) presents estimation results after adding *ruralpop*. The parameter estimates of the original variables are unaffected by this change and that for *ruralpop* negative and statistically significant ($p<0.05$). Columns (4)–(6) present results after incorporating *tobacco*, *logpm25*, and *education*, respectively. Notably, the parameter estimates for these new variables are not statistically significant while those for the original variables remain unchanged. On the other hand, the variable *ruralpop* loses statistical significance across these three estimations. Column (7) takes into account the introduction of *healthcare*, with none of the parameter estimates for *tobacco*, *logpm25*, *education*, and *healthcare* proving statistically significant. However, the coefficient estimate for *ruralpop* regains statistical significance ($p<0.05$).

Column (8) showcases the results of the full model following the inclusion of *corruption*. With the exception of *logpop*, *pop65plus*, *obesity*, and *ruralpop*, none of the parameter estimates for the included variables are statistically significant. Given the consistent lack of statistical significance for *tobacco*, *logpm25*, and *education*, these variables are subsequently removed, and the model is re-estimated in column (9). This column reveals estimation results after the exclusion of these variables. As observed in previous models, the coefficients for *logpop*, *pop65plus*, and *obesity* are positive and statistically significant ($p<0.001$). In addition, the coefficient estimate for *ruralpop* is negative and statistically significant ($p<0.01$). Lastly and notably, the coefficient estimates for *healthcare* and *corruption* are statistically significant, with positive and negative signs, respectively ($p<0.05$).

It is important to note that model accuracy, measured by the adjusted R^2^, significantly improved from 0.601 in column (1) to 0.672 with the introduction of *obesity*, with a minor increase to 0.68 after the addition of *ruralpop*. However, the adjusted R^2^ decreased to 0.656 after introducing *tobacco*, increased slightly to 0.658 with the inclusion of *logpm25*, and decreased significantly to 0.627 after adding *education*. Subsequently, the explanatory power increased significantly to 0.636 after introducing *healthcare* and further to 0.645 after introducing *corruption*. Remarkably, the last model, in column (9), which excludes the *tobacco*, *logpm25*, and *education* variables explains 69.1% of the variation in COVID-19 deaths, the highest among all nine estimations, indicating greater model accuracy and explanatory power.

A noteworthy observation from the estimation results is that the coefficient estimate for *healthcare* loses statistical significance in columns (7) and (8), suggesting a potential interaction effect with *tobacco*, *logpm25*, and *education*. This interaction could be attributed to how these variables influence healthcare expenditure. Nevertheless, the lack of statistical significance and lower adjusted R^2^ values imply that these variables do not contribute significantly to the model’s explanatory power.

In summary, focusing on the results in column (9), which show the best goodness-of-fit, it becomes apparent that countries with a larger proportion of people aged 65 and above experience higher COVID-19 mortality rates, and countries with higher obesity rates also report greater COVID-19 mortality. There is also evidence that countries with a greater proportion of people living in rural areas have lower COVID-19 mortality. In addition, countries with higher healthcare expenditure have higher COVID-19 mortality rates, while those with lower corruption levels, as suggested by a higher corruption perceptions index, have lower COVID-19 deaths. Lastly, contrary to prior research, there is no evidence of a connection between tobacco use, air pollution, education, and COVID-19 mortality.

### Robustness of the results

There are always concerns about potentially influential observations when using cross-country studies. This issue can be addressed using M-estimation with Huber weighting. M-estimation provides a robust method for estimating regression parameters, helping to address potential issues that might arise from the bootstrap resampling process. While bootstrapping is a powerful technique for estimating the sampling distribution of regression coefficients and assessing uncertainty, it can be sensitive to outliers in the data, potentially leading to biased or inefficient parameter estimates. M-estimation can mitigate the impact of outliers on their parameter estimates from its ability to assign lower weights to potential outliers, thereby reducing the latter’s influence on the final estimates and providing more reliable parameter estimates.

Table 5 summarizes the estimation results using the Huber M-estimator. Overall, the results remain largely consistent with those obtained using bootstrap linear regression. This reaffirms the previous findings even after accounting for potentially influential observations.

**Table 5:** Huber M-estimator estimation results (2020).

	(1)	(2)	(3)	(4)	(5)	(6)	(7)	(8)	(9)
logpop	0.976***	1.107***	1.101***	1.059***	1.049***	1.051***	1.042***	1.052***	1.070***
	(0.0663)	(0.0708)	(0.0698)	(0.0800)	(0.0835)	(0.0899)	(0.0920)	(0.0943)	(0.0777)
pop65plus	0.159***	0.142***	0.124***	0.108***	0.121***	0.110**	0.0925*	0.126**	0.132***
	(0.0209)	(0.0189)	(0.0208)	(0.0272)	(0.0339)	(0.0359)	(0.0387)	(0.0411)	(0.0283)
obesity		0.0894***	0.0758***	0.0831***	0.0834***	0.0735***	0.0712***	0.0705***	0.0755***
		(0.0127)	(0.0140)	(0.0165)	(0.0166)	(0.0176)	(0.0181)	(0.0185)	(0.0153)
ruralpop			− 0.0138*	− 0.0129	− 0.0131	− 0.0165	− 0.0182*	− 0.0216*	− 0.0184*
			(0.00688)	(0.00836)	(0.00843)	(0.00900)	(0.00910)	(0.00947)	(0.00753)
tobacco				0.0201	0.0186	0.0159	0.0184	0.0128	
				(0.0171)	(0.0172)	(0.0182)	(0.0186)	(0.0184)	
logpm25					0.177	0.134	0.260	0.159	
					(0.293)	(0.320)	(0.329)	(0.326)	
education						0.0388	0.00339	0.0717	
						(0.0896)	(0.0946)	(0.0947)	
healthcare							0.101	0.0942	0.116*
							(0.0715)	(0.0692)	(0.0573)
corruption								− 0.0243	− 0.0213*
								(0.0126)	(0.0100)
Intercept	− 10.34***	− 14.11***	− 13.03***	− 12.68***	− 13.18***	− 12.73***	− 13.30***	− 12.36***	− 12.20***
	(1.105)	(1.254)	(1.352)	(1.537)	(1.718)	(2.025)	(2.088)	(2.354)	(1.595)
Adj. $R^{2}$	0.582	0.659	0.670	0.641	0.638	0.612	0.614	0.636	0.682
$N$	174	163	163	141	141	129	127	124	150

Standard errors in parentheses

*$$p<0.05$$, **$$p<0.01$$, ***$$p<0.001$$.

**Table 6:** Subsamples estimation results (2020).

	Low to lower middle income			Upper middle income			High income		
	(1)	(2)	(3)	(1)	(2)	(3)	(1)	(2)	(3)
logpop	1.221***	1.152***	1.158***	0.969***	0.989***	1.022***	1.076***	1.092***	1.108***
	(0.192)	(0.135)	(0.142)	(0.208)	(0.138)	(0.135)	(0.202)	(0.149)	(0.197)
pop65plus	0.127	0.157	0.183	0.0570	0.0664	0.0947	− 0.0261	0.137*	0.129*
	(0.239)	(0.173)	(0.0965)	(0.109)	(0.0611)	(0.0552)	(0.0882)	(0.0673)	(0.0562)
obesity	0.0309	0.0449	0.0389	0.131**	0.122***	0.107**	0.0116	0.0772	0.0730*
	(0.0516)	(0.0342)	(0.0279)	(0.0467)	(0.0305)	(0.0326)	(0.0487)	(0.0426)	(0.0304)
ruralpop	− 0.0569**	− 0.0378**	− 0.0387**	− 0.00979	− 0.00585	− 0.00336	0.0134	− 0.00623	− 0.00263
	(0.0207)	(0.0130)	(0.0138)	(0.0220)	(0.0129)	(0.0143)	(0.0206)	(0.0201)	(0.0183)
tobacco	0.0118			0.0172			0.0567		
	(0.0380)			(0.0438)			(0.0444)		
logpm25	0.612			− 0.195			− 0.0786		
	(0.619)			(1.050)			(0.663)		
education	− 0.312			0.315			0.123		
	(0.234)			(0.208)			(0.202)		
healthcare	0.256	0.122	0.117	0.150	0.229*	0.261*	0.185	0.121	0.131
	(0.144)	(0.0733)	(0.0909)	(0.171)	(0.110)	(0.112)	(0.156)	(0.146)	(0.148)
corruption	0.0367	0.0111	0.0157	− 0.0761*	− 0.0524**	− 0.0512*	− 0.0364	− 0.0160	− 0.0120
	(0.0326)	(0.0200)	(0.0225)	(0.0321)	(0.0163)	(0.0226)	(0.0263)	(0.0215)	(0.0201)
Intercept	− 15.85***	− 13.42***	− 13.47***	− 10.52*	− 11.25***	− 11.93***	− 10.27*	− 13.62***	− 13.90***
	(3.186)	(2.313)	(2.504)	(5.341)	(2.516)	(3.100)	(4.302)	(3.462)	(3.647)
Adj. $R^{2}$	0.649	0.622	0.644	0.615	0.719	0.725	0.686	0.681	0.628
$N$	44	57	57	34	42	42	46	51	51

Standard errors in parentheses

*$$p<0.05$$, **$$p<0.01$$, ***$$p<0.001$$.

Another concern regarding the estimation results is the potential variation of the hypothesized relationships across different groups of countries. Given the substantial disparities among countries at various stages of development, the policy recommendations derived from the analysis may lack relevance and generalizability across all countries. One approach to address this heterogeneity is to estimate Eq. (1) for subsamples based on predetermined criteria. Utilizing the World Bank Atlas Method Gross National Income per capita (GNI) Operational Guidelines and Analytical Classifications for the Calendar Year 2019, countries are generally categorized as follows: low income if GNI $$\le \$1,035$$, lower middle income if $1035< GNI $$\le \$4,045$$, upper middle income if $4045< GNI $$\le \$12,535$$, and high income if GNI> $12,535.

Upon examination of the data, it is noted that the low income, lower middle income, upper middle income, and high income subsamples are too small to be estimated separately. As a result, the first two subsamples are merged to form a combined low income to lower middle income group when GNI $\le 4,045$ while the remaining subsamples are retained unchanged. All estimations are completed using Least Squares estimator with bootstrapped standard errors followed by M-estimation with Huber weighting and focusing on the parsimonious specification used in previous interpretations.

Table 6 summarizes the estimation results. Columns (1) present bootstrap estimation results for Eq. (1). Columns (2) display estimation results for the parsimonious specification that excludes *tobacco*, *logpm25*, and *education*. Columns (3) report Huber M-estimator estimation results for the parsimonious specification. For consistency, interpretations will focus on the results reported in columns (3).

First, the positive relationship between *pop65plus* and COVID-19 mortality, observed in the full sample, is found to be statistically significant only within the high-income group. Second, the observed positive relationship between *obesity* and COVID-19 mortality only holds within upper middle income and high-income groups. Third, the observed negative relationship between *ruralpop* and COVID-19 mortality only holds for low to lower middle income group. Fourth, The observed positive relationship between *healthcare* and COVID-19 mortality, as well as the negative relationship between *corruption* and COVID-19 mortality, only hold in the upper middle-income group.

An additional critical consideration for this study relates to the focus on the year 2020. While the examination of COVID-19 mortality in 2020 offers valuable insights into preparedness in initial responses and resource mobilization, the exclusion of subsequent years, notably 2021, could limit the assessment of policy effectiveness and healthcare system resilience. This is particularly important given that the year 2021 witnessed nearly double the number of deaths compared to the preceding year. To address this concern, all previous estimations in this study are completed using data from 2021. However, while most data sources are from the year 2021, exceptions include *logpm25* data, sourced from 2019, and data for *tobacco* and *healthcare*, which are from 2020. Summary statistics for all variables are reported in Table 7.

**Table 7:** Summary statistics.

Variable	Obs	Mean	Std. Dev.	Min	Max
logdeaths (2021)	174	7.6	2.411	2.197	13.054
logpop (2021)	200	15.345	2.454	9.324	21.069
pop65plus (2021)	200	9.597	6.816	1.396	35.97
obesity (2021)	186	23.14	13.09	1.86	74.94
ruralpop (2021)	200	38.198	23.559	0	86.544
tobacco (2020)	157	20.088	9.653	3.5	48.5
logpm25 (2019)	191	3.048	.685	1.717	4.421
education (2021)	139	4.495	2.044	1.424	15.585
healthcare (2020)	177	7.112	3.261	1.671	21.539
corruption (2021)	169	43.396	18.837	13	88

**Table 8:** Bootstrap estimation results (2021).

	(1)	(2)	(3)	(4)	(5)	(6)	(7)	(8)	(9)
logpop	0.858***	0.928***	0.931***	0.917***	0.934***	0.924***	0.924***	0.906***	0.885***
	(0.0592)	(0.0631)	(0.0671)	(0.0793)	(0.0779)	(0.0994)	(0.0934)	(0.109)	(0.0892)
pop65plus	0.119***	0.113***	0.119***	0.0734***	0.0583*	0.0530*	0.0535*	0.0644*	0.0915***
	(0.0163)	(0.0135)	(0.0138)	(0.0169)	(0.0226)	(0.0222)	(0.0242)	(0.0291)	(0.0232)
obesity		0.0709***	0.0748***	0.0766***	0.0758***	0.0922***	0.0922***	0.0883***	0.0780***
		(0.0113)	(0.0111)	(0.0114)	(0.0126)	(0.0128)	(0.0140)	(0.0169)	(0.0124)
ruralpop			0.00444	0.00194	0.00218	0.00580	0.00576	0.00322	
			(0.00513)	(0.00581)	(0.00605)	(0.00669)	(0.00684)	(0.00795)	
tobacco				0.0522***	0.0530***	0.0696***	0.0695***	0.0669***	0.0483***
				(0.0132)	(0.0131)	(0.0141)	(0.0147)	(0.0157)	(0.0129)
logpm25					− 0.228	− 0.0626	− 0.0658	− 0.126	
					(0.277)	(0.303)	(0.314)	(0.308)	
education						− 0.0171	− 0.0150	− 0.00695	
						(0.0940)	(0.0934)	(0.106)	
healthcare							− 0.00331	0.00268	0.0257
							(0.0479)	(0.0527)	(0.0387)
corruption								− 0.00969	− 0.0161
								(0.0139)	(0.00942)
Intercept	− 7.108***	− 9.665***	− 10.02***	− 10.20***	− 9.626***	− 10.65***	− 10.63***	− 9.659***	− 9.153***
	(0.965)	(1.076)	(1.175)	(1.387)	(1.778)	(1.999)	(1.825)	(2.669)	(1.565)
Adj. $R^{2}$	0.617	0.642	0.641	0.684	0.684	0.675	0.672	0.657	0.680
$N$	174	163	163	141	141	110	109	107	135

Standard errors in parentheses

*$$p<0.05$$, **$$p<0.01$$, ***$$p<0.001$$.

Table 8 reports estimation results for the year 2021 using bootstrap regression. Consistent with the previous findings, COVID-19 deaths are positively associated with the *pop65plus*, *obesity*, and *tobacco*. This latter variable did not have a statistically significant parameter estimate in the previous estimations. In addition and contrary to the previous estimations, there is no evidence of statistically significant links between *healthcare*, *corruption*, and COVID-19 mortality. These results are further reaffirmed in Table 9, which reports the Huber M-estimator regression estimation results.

**Table 9:** Huber M-estimator estimation results (2021).

	(1)	(2)	(3)	(4)	(5)	(6)	(7)	(8)	(9)
logpop	0.884***	0.959***	0.965***	0.953***	0.973***	0.991***	0.991***	0.986***	0.928***
	(0.0550)	(0.0595)	(0.0592)	(0.0596)	(0.0606)	(0.0671)	(0.0689)	(0.0769)	(0.0658)
pop65plus	0.120***	0.117***	0.125***	0.0817***	0.0582*	0.0553*	0.0551*	0.0577	0.0874**
	(0.0168)	(0.0153)	(0.0170)	(0.0196)	(0.0239)	(0.0242)	(0.0271)	(0.0311)	(0.0267)
obesity		0.0715***	0.0776***	0.0780***	0.0766***	0.0881***	0.0881***	0.0870***	0.0753***
		(0.0105)	(0.0116)	(0.0119)	(0.0117)	(0.0132)	(0.0134)	(0.0152)	(0.0108)
ruralpop			0.00729	0.00552	0.00594	0.00757	0.00758	0.00688	
			(0.00579)	(0.00620)	(0.00609)	(0.00656)	(0.00672)	(0.00761)	
tobacco				0.0494***	0.0513***	0.0618***	0.0619***	0.0611***	0.0476***
				(0.0128)	(0.0126)	(0.0135)	(0.0140)	(0.0152)	(0.0133)
logpm25					− 0.368	− 0.209	− 0.208	− 0.222	
					(0.213)	(0.233)	(0.243)	(0.260)	
education						0.00491	0.00515	0.0112	
						(0.0707)	(0.0780)	(0.0848)	
healthcare							0.00108	0.00321	0.0168
							(0.0581)	(0.0607)	(0.0454)
corruption								− 0.00265	− 0.0110
								(0.0107)	(0.00859)
Intercept	− 7.413***	− 10.07***	− 10.65***	− 10.86***	− 9.836***	− 11.10***	− 11.11***	− 10.86***	− 9.815***
	(0.920)	(1.061)	(1.150)	(1.148)	(1.250)	(1.477)	(1.563)	(1.948)	(1.250)
Adj. $R^{2}$	0.613	0.666	0.669	0.714	0.722	0.751	0.744	0.721	0.704
$N$	174	163	163	141	141	110	109	107	135

Standard errors in parentheses

*$$p<0.05$$, **$$p<0.01$$, ***$$p<0.001$$.

The final step in the addressing the concern related to excluding data for 2021 is completed by utilizing the World Bank Atlas Method Gross National Income per capita (GNI) Operational Guidelines and Analytical Classifications for the Calendar Year 2021. Countries are categorized as follows: low income if GNI $$\le \$1,085$$, lower middle income if $1,085< GNI $$\le \$4,255$$, upper middle income if $4,255< GNI $$\le \$13,205$$, and high income if GNI> $13,205. Consistent with the previous approach, the first two subsamples are merged to form a combined low income to lower middle income group when GNI $$\le 4,255$$ while the remaining subsamples remain unchanged.

**Table 10:** Subsamples estimation results (2021).

	Low to lower middle income			Upper middle income			High income		
	(1)	(2)	(3)	(1)	(2)	(3)	(1)	(2)	(3)
logpop	0.868***	0.881***	0.849***	0.431	0.645*	0.918***	1.018***	1.037***	0.993***
	(0.122)	(0.0930)	(0.125)	(0.613)	(0.256)	(0.0953)	(0.181)	(0.140)	(0.114)
pop65plus	0.194*	0.266**	0.244**	0.0728	0.0904	0.0595	− 0.00225	0.0545	0.0803
	(0.0819)	(0.0891)	(0.0764)	(0.138)	(0.0611)	(0.0458)	(0.0743)	(0.0414)	(0.0412)
obesity	0.0396	0.0508*	0.0348	0.118	0.0889	0.0349	0.0263	0.0532	0.0643***
	(0.0335)	(0.0234)	(0.0224)	(0.0873)	(0.0523)	(0.0231)	(0.0497)	(0.0275)	(0.0169)
ruralpop	− 0.00454			− 0.00854			0.0215		
	(0.0142)			(0.0442)			(0.0129)		
tobacco	0.0641**	0.0452**	0.0502*	0.0280	− 0.0200	− 0.00358	0.0804*	0.0754**	0.0517*
	(0.0215)	(0.0165)	(0.0203)	(0.0529)	(0.0323)	(0.0223)	(0.0362)	(0.0261)	(0.0231)
logpm25	− 0.424			0.678			0.721		
	(0.387)			(1.805)			(0.532)		
education	− 0.140			0.265			− 0.161		
	(0.156)			(0.390)			(0.196)		
healthcare	− 0.0548	0.00400	0.0256	0.00833	0.169	0.141	0.0934	0.00154	0.000141
	(0.138)	(0.0720)	(0.0783)	(0.235)	(0.112)	(0.0732)	(0.121)	(0.0727)	(0.0771)
corruption	0.0538*	0.0262	0.0277	− 0.0539	− 0.0173	0.000931	− 0.00652	− 0.0196	− 0.0102
	(0.0229)	(0.0187)	(0.0208)	(0.0673)	(0.0277)	(0.0179)	(0.0282)	(0.0159)	(0.0109)
Intercept	− 8.879***	− 10.84***	− 10.19***	− 3.063	− 4.512	− 7.974**	− 11.96*	− 10.67***	− 10.59***
	(2.394)	(1.936)	(2.421)	(12.43)	(4.068)	(2.293)	(5.455)	(2.796)	(1.896)
Adj. $R^{2}$	0.822	0.770	0.683	0.273	0.551	0.789	0.768	0.761	0.779
$N$	42	53	53	25	33	33	40	48	48

Standard errors in parentheses

*$$p<0.05$$, **$$p<0.01$$, ***$$p<0.001$$

Table 10 reports in Columns (1), (2), and (3) the estimation results of Eq. (1), the parsimonious specification that excludes *ruralpop*, *logpm25*, and *education* using bootstrap regression, and those of the parsimonious equation using the Huber M-estimator, respectively. It is worth noting that the parsimonious specification for 2021 differs from the one used with 2020 data due to the lack of statistical significance for *ruralpop* and presence of statistical significance for *tobacco*.

There are a number of observed differences in the presented estimation results. Contrary to previous findings that reported a positive relationship between *pop65plus* and COVID-19 mortality for the high-income group, the new estimations find that this relationship holds only for the low to lower middle-income groups. The new estimations also show the positive relationship between *obesity* and COVID-19 mortality holds only for the high-income group. Contrary to the previous estimations, there is a positive relationship between *tobacco* and COVID-19 mortality for the low to lower middle-income and high-income groups. Lastly, there is no link between *healthcare*, *corruption*, and COVID-19 mortality across any of the income groups.

## Discussion

This paper evaluates the determinants of COVID-19 mortality in 2020 using a sample of more than 140 countries with a specific focus on healthcare expenditure and corruption. Across the overall sample, the research reveals a positive association between COVID-19 deaths and several key factors: the proportion of the population aged 65 and older, the prevalence of obesity among adults, and healthcare expenditure. Conversely, it identifies a negative association between COVID-19 mortality and two factors: the proportion of the population living in rural areas and the corruption perceptions index. These findings highlight the heightened vulnerability of older individuals to severe COVID-19 outcomes, the established link between obesity and susceptibility to severe COVID-19 outcomes, and the connection between greater healthcare expenditure and corruption with higher COVID-19 mortality rates. Moreover, the study highlights disparities in mortality rates between rural and urban areas, with rural areas exhibiting lower mortality rates.

Upon further examination, with subsamples categorized according to countries’ levels of development, the study presents five key findings beyond the lack of statistically significant relationships between COVID-19 mortality, tobacco use, air pollution, and education. First, it finds that a higher proportion of the population aged 65 and older is significantly associated with greater COVID-19 deaths, particularly in high-income countries. This is likely due to the fact that the distribution of the population in high-income countries may include a disproportionately larger share of individuals aged 65 years and older compared to lower-income countries. This demographic skew toward older age groups would likely exacerbate the vulnerability to severe COVID-19 outcomes even in the presence of robust healthcare infrastructure.

Second, the prevalence of obesity within a population exhibits a positive and significant association with increased COVID-19 deaths, primarily in upper middle-income and high-income countries. This is likely due to the fact that in upper middle-income and high-income countries, obesity rates tend to be higher due to lifestyle factors and dietary habits.

Third, the analysis identifies disparities between rural and urban areas in low to lower middle income countries, with fewer deaths observed in countries with a greater share of the population residing in rural areas. This could be explained by the possibility that rural populations might have different social distancing practices or could be less densely populated, thereby reducing the spread of the virus and subsequently lowering mortality rates in these areas. However, it is important to consider that this disparity may also reflect the challenges faced by countries with limited resources in accurately tracking, recording, and reporting COVID-19-related deaths, particularly in rural settings.

Fourth, higher healthcare expenditure is found to be associated with higher COVID-19 mortality rates, albeit only in upper middle-income countries. This paper contends that greater healthcare expenditure likely reflects a country’s preparedness and ability to better track, document, and report COVID-19 deaths. This suggests that countries with low expenditure on health care may not have the resources to accurately report COVID-19 deaths and may even have an incentive to under-report. It is important to note, however, that while a high expenditure share on healthcare could suggest a large healthcare sector, it is not indicative of the quality of healthcare. For instance, based on World Bank data, the United States spent more than 16.67% of its GDP on health care (second to Vanuatu) in 2019 and managed to get the world’s highest number of COVID-19 deaths in 2020 in absolute terms (352,000) and the 15th largest in per capita terms. Most European countries that have relatively high expenditure on health care also happen to have a high number of deaths in absolute and in per capita terms. Another important observation is that several countries that have low healthcare expenditure have reported a relatively low number of deaths. In such countries, extreme lockdown and confinement measures would have been warranted after the realization that the pandemic would be too overwhelming and could lead to greater mortality and a collapse of the healthcare system. Of course, fewer reported deaths could also be the result of insufficient resources to track and report all deaths related to COVID-19.

Fifth, higher levels of corruption are linked to more COVID-19 deaths, particularly in upper middle-income countries. While this finding may seem counterintuitive at first glance, given that corruption is often more prevalent in low-income countries, there are a number of plausible explanations. For instance, corruption in upper middle-income countries may involve more complex networks and higher levels of collusion between government officials, private sector actors, and other stakeholders. These corruption networks could have serious implications for healthcare delivery and pandemic response, leading to systemic weaknesses that contribute to higher COVID-19 mortality rates. In contrast, corruption in low-income countries may be more localized or decentralized, with fewer layers of bureaucracy, making it less directly linked to mortality outcomes.

These findings gain added significance with the addition of an analysis using data for 2021. First, the fact that a higher proportion of the population aged 65 and older is significantly associated with greater COVID-19 deaths in the low to lower-middle-income groups rather than in the high-income group could be explain by one key factor. In 2021, high-income countries generally have more robust healthcare systems and better access to medical resources, including critical care facilities, advanced treatments, greater access to vaccines and resources for vaccine distribution, allowing them to implement more effective public health measures and containment strategies to protect older populations from COVID-19, such as early lockdowns, widespread testing, contact tracing, and prioritizing older populations for vaccination. These measures could have led to a more significant reduction in COVID-19 mortality rates among older adults in high-income countries compared to low to lower-middle-income countries.

Second, the positive relationship between the prevalence of obesity within a population and COVID-19 mortality reaffirms the previous findings albeit only for high-income countries, which are known to have the highest rates of obesity. Third, the emergence of a positive relationship between the prevalence of tobacco use and COVID-19 mortality can be explained by the fact that although the prevalence of tobacco use has decreased on average between 2019 and 2021, as shown in Tables 2 and 7, the detrimental health effects of tobacco use, including its impact on respiratory health and immune function, often accumulate over time.

Fourth and more importantly, the absence of evidence linking COVID-19 mortality to healthcare expenditure and corruption is not only compelling but also reassuring. This finding implies that countries may have undertaken substantial investments to strengthen their healthcare infrastructure, expand testing and treatment capabilities, and enhance access to medical resources for COVID-19 patients. These proactive measures, spurred by the exigencies of the pandemic, likely contributed to a convergence in preparedness levels across countries and may have curtailed the impact of corruption on pandemic outcomes. This implies that, when confronted with public health crises as severe as the COVID-19 pandemic, countries may not be unduly constrained by their level of development in mobilizing resources.

This paper faces some limitations, which should be addressed in future research. First, while statistical associations are established, none imply causation. Second, the positive link between healthcare expenditure and COVID-19 mortality may be influenced by unaccounted confounding variables. Third, the study does not evaluate the unique circumstances of individual countries, such as variations in healthcare system structures, the timing of the pandemic’s arrival, and the effectiveness of public health measures. Fourth, the analysis also does not comprehensively evaluate the unique circumstances of individual countries, which could significantly influence COVID-19 mortality. Variations in healthcare system structures, including differences in healthcare infrastructure, staffing levels, and access to medical resources, may have a substantial impact on a country’s ability to effectively respond to the pandemic. Fifth, the study’s findings may be subject to measurement limitations and data quality issues inherent in cross-national datasets.

This research has important policy implications. Given the vulnerability of older individuals to public health crises such as pandemics, especially in low to lower-middle-income countries, policymakers should prioritize targeted protection measures for this demographic group. This includes ensuring equitable access to vaccines, implementing early containment strategies, and strengthening healthcare systems to provide adequate support and care for older populations. In high-income countries, proactive public health interventions should be adopted to tackle the obesity crisis, especially given its exacerbating impact on the COVID-19 pandemic. Beyond initiatives to improve nutrition, promote physical activity, and combat sedentary behaviors, policymakers should consider using measures such as a fat tax or an outright ban of foods that are deemed unhealthy. Policy interventions should also raise awareness about the risks of COVID-19 and similar pandemics for tobacco users and encourage individuals to quit smoking. Policymakers could approach this issue more proactively by strengthening tobacco control policies and regulations to reduce tobacco use prevalence and protect public health. This includes measures such as an outright ban, tobacco taxation, smoke-free laws, restrictions on tobacco advertising and promotion, and the introduction and enforcement of age restrictions for tobacco sales.

## Data Availability

The study made use of publicly-available country-level data, which do not involve experiments or human subjects. They are available upon reasonable request.
